# A Cross-Sectional Study of Retired Great British Olympians (Berlin 1936–Sochi 2014): Olympic Career Injuries, Joint Health in Later Life, and Reasons for Retirement from Olympic Sport

**DOI:** 10.1186/s40798-021-00339-1

**Published:** 2021-07-31

**Authors:** Dale J. Cooper, Mark E. Batt, Mary S. O’Hanlon, Debbie Palmer

**Affiliations:** 1 School of Allied Health Professions, Keele University, Keele, Staffordshire UK; 2 Centre for Sport, Exercise and Osteoarthritis Research Versus Arthritis, Nottingham University Hospitals NHS Trust, Nottingham, UK; 3 Academic Department of Orthopaedics, Trauma and Sports Medicine, University of Nottingham, Nottingham, UK; 4 Institute for Sport, PE and Health Sciences Moray House School of Education and Sport, University of Edinburgh, Edinburgh, UK

**Keywords:** Prevalence, Injury, Pain, Osteoarthritis, Olympians

## Abstract

**Background:**

The relationship between Olympic career sport injury and the long-term musculoskeletal health of the elite athlete remains unclear. This study describes the lifetime prevalence of medical attention injuries that occurred during training and/or competition as part of the athlete’s Olympic career, reasons for retirement from Olympic sport, and the point prevalence of pain and osteoarthritis (OA) among retired Great Britain’s (GB) Olympians.

**Methods:**

This cross-sectional study involved distributing a questionnaire to retired GB Olympians who had competed at 36 Olympic Games between Berlin 1936 and Sochi 2014. The questionnaire captured Olympic career injury history (lasting ≥ 1 month), sport exposure, musculoskeletal pain (last 4 weeks), physician-diagnosed OA, and joint replacement. Injury prevalence was calculated for sports with a minimal of 15 respondents. Adjusted odds ratios (aOR) were estimated in logistic regression for pain, OA, and joint replacement. Models were adjusted for age, sex, BMI, and career duration.

**Results:**

Six hundred fifty (57.8% male; 42.2% female) retired athletes representing 40 sports (29 summer; 11 winter), aged 60.5 years (range 23–97), completed the questionnaire. Overall, 721 injuries (368 athletes) were self-reported equating to a lifetime Olympic career injury prevalence of 56.6%. Injury prevalence was highest in field athletics (81.0%), gymnastics (75.0%), and track athletics (67.7%). Injuries most frequently occurred at the knee (19.0%), lower back (15.4%), and shoulder (11.5%). Of those injured, 19.5% retired from sport due to injury. Pain was most prevalent at the lumbar spine (32.8%), knee (25.3%), and hip (22.5%), and OA at the knee (13.4%), hip (10.4%), and lumbar spine (4.6%). Injury was associated with pain at the hip (aOR 4.88; 95% CI, 1.87–12.72, p = 0.001), knee (aOR 2.35; 95% CI, 1.45–3.81, p = 0.001), and lumbar spine (aOR 2.53; 95% CI, 1.63–3.92, p < 0.001); OA at the hip (aOR 5.97; 95% CI, 1.59–22.47, p = 0.008) and knee (aOR 3.91; 95% CI, 2.21–6.94, p < 0.001); and joint replacement at the hip (aOR 8.71; 95% CI, 2.13–35.63, p = 0.003) and knee (aOR 5.29; 95% CI, 2.39–11.74, p < 0.001).

**Conclusion:**

The lifetime prevalence of Olympic career injury was 56.6%, with those injured more likely to self-report current pain and/or OA at the hip, knee, and lumbar spine and joint replacement at the hip and knee.

## Key Points

- Medical attention injuries (lasting > 1 month) were sustained by 56.6%; injuries occurred most frequently at the knee, lumbar spine, and shoulder with 19.5% of those injured forced to retire early.
- Fractures, dislocations, and head injuries occurred more frequently in winter sports; females were more at risk of stress fractures, and injuries were associated with pain and osteoarthritis in later life.
- Prevention strategies should aim to reduce injuries in the primary weight-bearing joints of the lower limbs to mitigate the risk of early onset of osteoarthritis and joint replacement in retirement.

## Background

A key mandate of the International Olympic Committee (IOC) is to encourage and support measures that protect the health of the athlete in sport [1, 2]. The IOC has promoted epidemiological studies for identifying and reducing injuries and maximising athlete health [1]. The monitoring of injuries was implemented for the first time at the 2008 Beijing Olympic Games [3], and the surveillance of both injuries and illnesses was subsequently introduced at the 2010 Vancouver Olympic Games [4]. Published literature from injury surveillance studies have provided a description of the types and mechanisms of injuries sustained in current elite athletes on a seasonal basis and during major sporting events [5–10]. However, the relationship between sport injury and the long-term musculoskeletal health of the elite athlete remains unclear.

Previous studies have indicated, compared with population controls, that former elite athletes have a lower risk of stroke, cancer, diabetes, heart disease, and mortality in later life [11–13]. Whereas the degree of musculoskeletal morbidity has been shown to be higher in former male athletes from Olympic sports [14], soccer [15], cricket [12], and Rugby Union [13]. Musculoskeletal injuries are common in elite athletes and these can lead to early post-traumatic osteoarthritis (PTOA) [16, 17]. Post-traumatic OA progresses more rapidly to end-stage disease compared to idiopathic OA and can impair health-related quality of life [16, 18]. To date, retired-athlete studies have investigated and reported an association between injury with OA but these studies have focused on male athletes and at isolated joints [14, 15, 19].

To our knowledge, no epidemiological studies have accounted for the occurrence of lifetime career injuries and the prevalence of pain and OA across multiple body sites in a retired Olympic male and female athletic population. If we are to prevent injury, premature retirement, and the sequelae of joint disease in retired elite athletes, knowledge is required of the nature of lifetime and career-ending injuries, the joints most commonly affected by joint disease, and the nature of injuries that occurred at those joints. The aims of this study are to describe the lifetime prevalence of medical attention injuries that occurred during training and/or competition as part of the athlete’s Olympic career, the reasons for retirement from Olympic sport, and the point prevalence of pain and OA among retired Great Britain’s (GB) Olympians.

## Methods

### Study Design

This cross-sectional study involved distributing a letter by post or email inviting GB Olympians the opportunity to complete and return a postal questionnaire or the option of completing an online version. The questionnaire was distributed to both current and retired athletes registered on the British Olympic Association (BOA) database. For the present study, we excluded responses from those who were training to qualify for or compete at any upcoming Olympic Games. The first phase of data collection took place between May 2014 and April 2015 and involved distributing the questionnaire to those living in the UK and to those residing overseas. Two email reminders and one postal reminder were sent to non-responders. The second phase of data collection involved the BOA Athlete’s Commission distributing individual copies of the questionnaire. All questionnaires were returned by December 2016.

### Data Collection and Management

The study questionnaire content is reported elsewhere [20], and data collected on demographics, sports career participation, medical history, musculoskeletal health, and Olympic career injury are presented in this study. Baseline questions captured self-reported information on age (years), sex, height (cm), weight (kg), and ethnicity. Early life (i.e. during the 20s) and current height and weight data were self-reported and used to calculate Olympic career and current body mass index (BMI kg/m^2^) separately. Sport participation included the frequency and duration for the period of training and competition leading up to their first Olympic Games until retirement from their last Olympic Games. The presence of a significant injury was determined by asking participants: “have you ever sustained a significant injury that caused pain for most days during a 1-month period or more and for which you consulted a medical professional or a health provider such as a general practitioner?” [20]. Injury data was collected in line with IOC injury surveillance methods and included anatomical location, type, mechanism of injury, competition, and training injuries [8, 9, 21, 22]. Participants were asked to self-report the location and duration of current pain within the last month [23]. A body manikin was used to self-report the location of pain using a method shown to be repeatable [24]. Injuries and bodily pain lasting less than 1 month were not recorded and non-sport injuries were excluded. The presence of OA was determined by asking participants: “have you ever been diagnosed with OA in any of your joints by a physician, and if so, please state which joint/s?” Participants were asked to record the date, type, and reasons for joint replacement surgery. Where GB Olympians had competed in at least two disciplines at Olympic level, preference was given to the discipline in which the participant had spent the longest time competing.

### Definitions

A consensus meeting took place with 14 retired GB Olympic athletes at the BOA to establish agreement for defining Olympic career injury and retirement from Olympic sport. A retired Olympian was defined as ‘an Olympian who was retired from Olympic competition (i.e. those who had confirmed they had retired and were no longer training to qualify for or compete at any upcoming Olympic Games)’. A significant Olympic career injury was adapted from the IOC approach for recording injury in multi-sport events [22]. Injury was defined as ‘an injury that required medical attention irrespective of absence from competition or training and occurred during training for, and/or competition, as part of the athlete’s Olympic career and caused pain for most days during a 1-month period or more’. In agreement with consensus statements, recurrent injury was defined by the same location and type, which occurred after an athlete returned to full participation from the previous injury [22, 25].

### Statistical Analysis

Statistical analyses were conducted within SPSS 25.0 (SPSS Inc., Chicago, IL, USA). Descriptive statistics are presented as frequencies (proportion) for categorical variables, and for numerical variables data are presented with the mean and standard deviation, or median and range where data are not normally distributed. The prevalence of career injury was calculated by dividing the number of injured athletes by the total number of athletes and presented as percentages (%) with 95% confidence intervals (CIs). Crude odds ratios with 95% CIs were estimated in logistic regression for factors and outcomes of hip and knee replacement. These models were adjusted with staged adjustment for the potential confounders of age, sex, BMI, injury, and Olympic career exposure. The most severe hip and knee joint were selected as the index joint for analysis. Significant injuries were matched according to the index joint and included if they preceded the date of OA diagnosis and joint replacement. Significance was accepted at p < 0.05 and imputation was not undertaken for missing values.

### Power Calculation

The power calculation was performed using GPower V.3.1.9.2. In a sample of 650, with a 1:1 ratio for exposed and unexposed, we had at least 80% power to detect an odds ratio of 1.96 and 2.16 or greater at 5% significance based on a prevalence rate of 7% and 5% for hip and knee replacement one-tailed (assuming those with a joint injury would have greater risk), respectively.

### Research Ethics Approval

This study was approved by the University of Nottingham Research Ethics Committee (Reference No: K13022014). All procedures involving research participants were in accordance with the ethical standards of the university institution review board and with the 1964 Helsinki Declaration and its later amendments. All participants received written information at the start of the study, detailing how data would be stored and treated confidentially, ensuring athlete anonymity at all times. It was made explicitly clear to participants that by completing and returning a questionnaire they gave implied consent for their data to be used anonymously for the purposes of this study.

## Results

### Olympian Characteristics

**Fig. 1:**
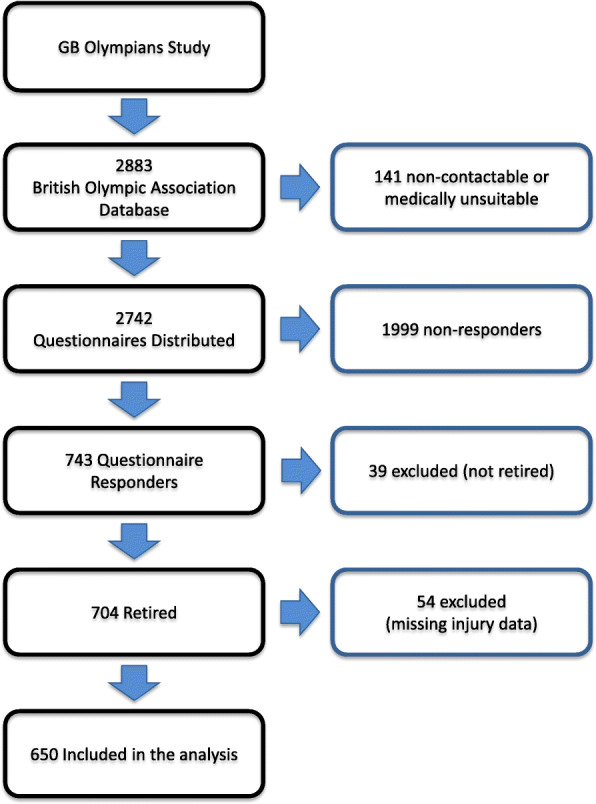
Flow chart describing the recruitment of retired Great Britain’s (GB) Olympic athletes included in this study. The flow chart describes those who could not be contacted or were medically unsuitable, the number of questionnaires distributed, the number of questionnaires returned, and the number of questionnaires included in the analysis. GB Olympic athletes who did not respond or were ineligible for the study were excluded.

A total of 2742 questionnaires were distributed to current and retired athletes and 743 returned a completed questionnaire (27.1% response rate). Of the 743 returns achieved, 650 were retired from their Olympic career and had data for the prevalence analysis of injury (Fig. 1). Table 1 reports the characteristics of the respondents (male 57.8%; female 42.2%) that were aged between 23 and 97 years (median 60.5 years). Respondents had competed (mean 9.8 ± 6.2 years) in at least one of 40 Olympic sports (Table 2). Of the 650 respondents, 66 had competed in 11 sports at the Winter Olympics and 584 had competed in 29 sports at the Summer Olympics. All participants were retired (mean 31.2 ± 16.7 years) from their Olympic careers and participation spanned Summer and Winter Olympic Games from Berlin in 1936 to Sochi in 2014.

**Table 1:** Olympian characteristics

	All, n = 650	Female, n = 274	Male, n = 376	*P* values
Anthropometrics				
Age (years), median (range)	60.5 (23–97)	54 (23–93)	66 (26–97)	**p < 0.001**
Height (cm), mean (SD)	175.6 (10.2)	169.0 (7.6)	180.4 (9.0)	**p < 0.001**
Weight (kg), mean (SD)	76.6 (15.8)	67.8 (13.8)	83.1 (14.0)	**p < 0.001**
Current BMI (kg/m^2^), mean (SD)	24.7 (4.0)	23.7 (4.7)	25.4 (3.3)	**p < 0.001**
Olympic career BMI (kg/m^2^), mean (SD)	22.8 (2.9)	21.7 (2.5)	23.5 (2.9)	**p < 0.001**
Sport exposure				
Athletes by summer/winter sport, n (%)	274 (42.2) 376 (57.8)	250 (91.2) 24 (8.8)	334 (88.8) 42 (11.2)	p = 0.382
Number of international years doing Olympic sport, mean (SD)	9.8 (6.2)	10.2 (6.8)	9.4 (5.7)	p = 0.301
Number of years retired from Olympic participation, mean (SD)	31.2 (16.7)	27.5 (16.2)	34.2 (16.5)	**p < 0.001**

A comparison for differences between the groups was analysed using the unpaired t test for continuous variables, or Mann-Whitney U test where appropriate, and categorical variables were analysed using *χ*^2^. Statistically significant differences are highlighted in bold

**Table 2:** Injuries by Olympic sport and sex in retired Great Britain’s Olympians

	Athletes (n; m/f)*	Injuries (n)	Training/competition/unknown** (%)	Injuries per athlete (mean n)	Injured athletes (n; m/f)		Injury prevalence (95% CI)	Exact 95% CI***
Summer sports								
Archery	10 (3/7)	3	0/66.7/33.3	0.30	3 (1/2)		–	
Athletics (track)	99 (53/46)	128	62.5/28.9/8.6	1.29	67 (35/32)		67.7%	58.3 to 77.1
Athletics (field)	21 (8/13)	29	37.9/48.3/13.8	1.38	17 (7/10)		81.0%	62.6 to 99.3
Athletics (road)	19 (12/7)	30	53.3/43.3/3.4	1.58	10 (7/3)		52.6%	27.9 to 77.4
Aquatics								
Swimming	73 (26/47)	58	50/25.9/24.1	0.79	34 (8/26)		46.6%	34.9 to 58.3
Diving	13 (4/9)	17	58.8/23.5/17.7	1.31	8 (1/7)		–	
Syn. swimming	3 (0/3)	5	100/0/0	1.67	2 (0/2)		–	
Water polo	4 (3/1)	6	100/0/0	1.50	2 (1/1)		–	
Badminton	4 (2/2)	4	75/0/25	1.00	2 (2/0)		–	
Basketball	4 (3/1)	6	33.3/50/16.7	1.50	3 (2/1)		–	
Boxing	6 (6/0)	1	0/0/100	0.17	1 (1/0)		–	
Canoeing	28 (17/11)	13	76.9/7.7/15.4	0.46	8 (3/5)		28.6%	10.7 to 46.4
Cycling	23 (17/6)	28	25/67.9/7.1	1.22	15 (10/5)		65.2%	44.2 to 86.3
Equestrian	10 (3/7)	15	26.7/60/13.3	1.50	5 (2/3)		–	
Fencing	23 (13/10)	18	33.3/55.6/11.1	0.78	12 (6/6)		52.2%	30.1 to 74.3
Football	5 (5/0)	5	0/80/20	1.00	4 (4/0)		–	
Gymnastics	20 (9/11)	31	64.5/32.3/3.2	1.55	15 (7/8)		75.0%	54.2 to 95.8
Handball	1 (1/0)	0	–	0.00	–		–	
Hockey	57 (46/11)	62	25.8/61.3/12.9	1.09	33 (26/7)		57.9%	44.7 to 71.1
Judo	10 (6/4)	26	46.2/50/3.8	2.60	7 (4/3)		–	
Modern pentathlon	7 (5/2)	11	54.5/36.4/9.1	1.57	6 (4/2)		–	
Rowing	93 (54/39)	93	67.7/21.5/10.8	1.00	50 (26/24)		53.8%	43.4 to 64.1
Sailing	20 (15/5)	20	45/25/30	1.00	10 (5/5)		50.0%	26.0 to 74.0
Shooting	10 (6/4)	10	30/70/0	1.00	7 (4/3)		–	
Table tennis	1 (1/0)	2	0/100/0	2.00	1 (1/0)		–	
Taekwondo	1 (0/1)	0	–	0.00	–		–	
Tennis	5 (4/1)	7	14.3/85.7/0	1.40	2 (1/1)		–	
Volleyball	2 (2/0)	3	100/0/0	1.50	1 (1/0)		–	
Weightlifting	8 (6/2)	19	47.4/42.1/10.5	2.38	7 (5/2)		–	
Wind surfing	1 (1/0)	1	0/0/100	1.00	1 (1/0)		–	
Wrestling	3 (3/0)	4	25/50/25	1.33	3 (3/0)		–	
**Winter sports**							–	
Alpine skiing	12 (7/5)	14	6/5/3	1.17	2 (2/0)		–	
Biathlon	4 (4/0)	4	50/50/0	1.00	2 (2/0)		–	
Bobsleigh	15 (13/2)	13	53.8/23.1/23.1	0.87	9 (7/2)		60.0%	31.9 to 88.1
Cross-country skiing	9 (6/3)	11	18.2/81.8/0	1.22	3 (3/0)		–	
Figure skating	10 (2/8)	5	100/0/0	0.50	5 (1/4)		–	
Ice hockey	1 (1/0)	1	0/100/0	1.00	1 (1/0)		–	
Luge	3 (3/0)	3	33.3/66.7/0	1.00	2 (2/0)		–	
Short tr. sp. skating	7 (3/4)	7	85.7/14.3/0	1.00	5 (2/3)		–	
Skeleton	1 (0/1)	1	100/0/0	1.00	1 (0/1)		–	
Skiing: freestyle	2 (1/1)	7	57.1/42.9/0	3.50	2 (1/1)		–	
Speed skating	2 (2/0)	0	–	–	–		–	
Total summer sports	584 (334/250)	655	50.7/37.6/11.7	1.12	336 (178/158)		57.5%	53.5 to 61.6
Total winter sports	66 (42/24)	66	51.5/39.4/9.1	1.00	32 (21/11)		48.5%	36.1 to 60.9
**Overall total**	**650**	**721**	**50.8/37.7/11.5**	**1.11**	**368 (199/169)**		56.6%	52.8 to 60.4

*m males, *f* females

**Occurred in training, competition, unknown (i.e. competition or training)

***CI confidence intervals; prevalence not reported for sports with < 15 Olympians

### Prevalence of Injury

There were 721 injuries (368 athletes) reported from 650 retired GB Olympians equating to 56.6% (95% CI 52.8–60.4) of Olympians reporting at least one significant Olympic career-related injury. Each Olympian reported a mean of 1.11 injuries (female = 1.35; male = 0.94) during their Olympic career, with 50.8% (n = 366) and 37.7% (n = 272) of injuries attributed to training and competition, respectively (Table 2). Injury prevalence was 48.5% (36.1–60.9) for winter Olympians compared with 57.5% (53.5–61.6) for summer Olympians. After adjustment (age, sex, BMI, and career duration), there was no significant difference in the number of injured athletes competing at the Winter Olympics compared to the Summer Olympics (aOR 1.00; 95% CI, 0.98–1.02, p = 0.956). However, participants from weight-bearing sports were more likely to self-report a significant injury (aOR 1.69; 95% CI, 1.22, 2.36, p = .002). By sport, injury prevalence was highest in field athletics (81.0% (62.6–99.3)), gymnastics (75.0% (54.2–95.8)), track athletics (67.7% (58.3–77.1)), and lowest for canoeing (28.6% (10.7–46.4)), swimming (46.6% (34.9–58.3)), and sailing (50.0% (26.0–74.0)) (sports with n ≥ 15 participants).

### Injury Location, Type, and Cause

Injuries most frequently occurred at the knee (19.0% (137/721)), lower back (15.4% (111/721)), and shoulder (11.5% (83/721)) (Table 3). The most common injury types were ligament sprain (19.3% (139/721)), traumatic fractures (18.4% (133/721)), and muscle injury (12.2% (88/721)) (Table 4). Traumatic fractures accounted for 22.2% (78/352) of all injuries in males and 14.9% (55/369) in females; stress fractures accounted for 3.1% (11/352) of all injuries in males and 6.8% (25/369) in females. Females were no more likely to report any injury (aOR 0.84; 95% CI, 0.59–1.18, p = 0.32) or traumatic fracture than males (aOR 1.33; 95% CI, 0.82–2.16, p = 0.243); however, females were more likely to report a stress fracture (aOR 2.85; 95% CI, 1.15–7.04, p = 0.023). There was a single catastrophic injury with spinal cord injury and paraplegia as the outcome.

**Table 3:** Location of injuries (%) in retired Great Britain's Olympians

Body part	Number of female injuries (%)	Number of male injuries (%)	Total number of injuries (%)
Knee	69 (18.7)	68 (19.3)	137 (19.0)
Lower back	61 (16.5)	50 (14.2)	111 (15.4)
Shoulder	33 (8.9)	50 (14.2)	83 (11.5)
Ankle	38 (10.3)	29 (8.2)	67 (9.3)
Others/unknown	28 (7.6)	27 (7.7)	55 (7.6)
Lower leg	21 (5.7)	27 (7.7)	48 (6.7)
Foot/toe	21 (5.7)	11 (3.1)	32 (4.4)
Thigh	11 (3.0)	15 (4.3)	26 (3.6)
Achilles ten.	13 (3.5)	13 (3.7)	26 (3.6)
Wrist	14 (3.8)	11 (3.1)	25 (3.5)
Elbow	16 (4.3)	4 (1.1)	20 (2.8)
Head	6 (1.6)	10 (2.8)	16 (2.2)
Hip	6 (1.6)	7 (2.0)	13 (1.8)
Sternum/rib	8 (2.2)	5 (1.4)	13 (1.8)
Neck	5 (1.4)	5 (1.4)	10 (1.4)
Upper back	6 (1.6)	4 (1.1)	10 (1.4)
Forearm	5 (1.4)	3 (0.9)	8 (1.1)
Finger	3 (0.8)	3 (0.9)	6 (0.8)
Hand	2 (0.5)	3 (0.9)	5 (0.7)
Pelvis/SIJ	2 (0.5)	1 (0.3)	3 (0.4)
Upper arm	–	3 (0.9)	3 (0.4)
Abdomen	–	2 (0.6)	2 (0.3)
Thumb	1 (0.3)	1 (0.3)	2 (0.3)
Total	369	352	721

**Table 4:** Injury type (%) in retired Great Britain’s Olympians

Type of injury	Injuries in female (%)	Injuries in male (%)	Total number of injuries (%)
Ligament injury (sprain)	84 (22.8)	55 (15.6)	139 (19.3)
Fracture (traumatic)	55 (14.9)	78 (22.2)	133 (18.4)
Muscle injury (strain)	44 (11.9)	44 (12.5)	88 (12.2)
Tendinopathy	33 (8.9)	44 (12.5)	77 (10.7)
Cartilage injury	25 (6.8)	27 (7.7)	52 (7.2)
Disc prolapse	24 (6.5)	16 (4.5)	40 (5.5)
Dislocation/subluxation	15 (4.1)	22 (6.3)	37 (5.1)
Stress fracture	25 (6.8)	11 (3.1)	36 (5.0)
Nerve injury	7 (1.9)	5 (1.4)	12 (1.7)
Contusion/hematoma	8 (2.2)	1 (0.3)	9 (1.2)
Compartment syndrome	–	4 (1.1)	4 (0.6)
Fasciitis	3 (0.8)	–	3 (0.4)
Laceration	2 (0.5)	1 (0.3)	3 (0.4)
Muscle cramps	2 (0.5)	1 (0.3)	3 (0.4)
Concussion	2 (0.5)	–	2 (0.3)
Impingement	–	2 (0.6)	2 (0.3)
Dental injury	–	1 (0.3)	1 (0.1)
Amputation	–	1 (0.3)	1 (0.1)
Medial tibial stress syndrome	1 (0.3)	–	1 (0.1)
Other/missing	39 (10.6)	39 (11.1)	78 (10.8)
Total	369	352	721

The most prominent injury locations in GB Olympians who had participated in the Summer Olympics were the knee (18.6% (122/655)), lower back (15.7% (103/655)), and shoulder (10.8% (71/655)), and this compares to the knee (22.7% (15/66)), shoulder (18.2% (12/66)), and lower back (12.1% (8/66)) among those who had participated in the Winter Olympics. The number of sport-related injuries in the Summer and Winter Olympics were comparable: competition 37.6% (246/655) versus 39.4% (26/66) and training 50.7% (332/655) versus 51.5% (34/66), respectively (Table 2). The most common reported injury mechanisms were non-contact trauma 40.3% (85/211), overuse gradual onset 30.3% (64/211), and sudden onset 16.6% (35/211).

### Reasons for Retirement from Olympic Career

Of the participants included in this study, 19.5% (127/650) reported they had retired early from their Olympic careers because of injury. Of those forced to retire due to injury, 32.3% (41/127) had suffered a recurrent injury and 26.0% (33/127) reported retiring due to a one-off injury. The main locations of injuries responsible for retirement from sport were the lumbar spine (26.0% (33/127)), knee (24.4% (31/127)), ankle (14.2% (18/127)), lower leg (8.7% (11/127)), shoulder (7.1% (9/127)), and hip (5.5% (7/127)). The main types of injury responsible for early retirement from sport were ligament injury (19.7% (25/127)), intervertebral disc prolapse (18.1% (23/127)), tendinopathy (14.2% (18/127)) cartilage tears (9.4% (12/127)), and fracture +/- dislocation (5.5% (7/127)). Approximately 80.5% (523/650) of all participants retired for non-injury reasons: because they had achieved all that was possible (37.9% (198/523)), chosen an alternative career (27.9% (146/523)), declined in fitness (12.6% (66/523)), financial reasons, or starting a family (9.0% (47/523)).

### Musculoskeletal Health

The prevalence of pain was highest at the lumbar spine (32.8% (197/600)), knee (25.3% (152/600)), and hip (22.5% (135/600)), and OA was most prevalent at the knee (13.4% (85/635)), hip (10.4% (66/635)), and lumbar spine (4.6% (29/635)) (Table 5). The prevalence of hip and knee replacement resulting from end-stage OA was 7.0% (44/628) and 5.4% (34/628). After adjustment (age, BMI, sex, career duration), injury was associated with pain at the hip (aOR 4.88; 95% CI, 1.87–12.72, p = 0.001), knee (aOR 2.35; 95% CI, 1.45–3.81, p = 0.001) and lumbar spine (aOR 2.53; 95% CI, 1.63–3.92, p < 0.001), and OA at the hip (aOR 5.97; 95% CI, 1.59–22.47, p = 0.008) and knee (aOR 3.91; 95% CI, 2.21–6.94, p < 0.001). Those injured at the hip, knee, or ankle were more likely to self-report physician-diagnosed OA in these joints before 45 years [aOR 4.28; 95% CI, 1.08–17.02, p = 0.039]. Injury was the main attributable factor associated with joint replacement at the hip (aOR 8.71; 95% CI, 2.13–35.63, p = 0.003) and knee (aOR 5.29; 95% CI, 2.39–11.74, p < 0.001). A one-unit increase in age was associated with joint replacement at the hip (aOR 1.09, 95% CI, 1.06–1.12, p < 0.001) and knee (aOR 1.07; 95% CI, 1.04–1.10, p < 0.001), with BMI associated with knee replacement (aOR 1.11; 95% CI, 1.03–1.20, p = 0.004) (Table 6).

**Table 5:** Musculoskeletal health in retired Great Britain’s Olympians

	Women (n = 246)	Prevalence % (95% CI)	Men (n = 354)	Prevalence % (95% CI)	All (n = 600)	Overall prevalence (95% CI)
**Pain (last 4 weeks)**						
Lumbar spine	83	33.7 (27.8–39.7)	114	32.2 (27.3–37.1)	197	32.8 (29.1–36.6)
Knee	65	26.4 (20.9–32.0)	87	24.6 (20.1–29.1)	152	25.3 (21.8–28.8)
Hip	51	20.7 (15.6–25.8)	84	23.7 (19.3–28.2)	135	22.5 (19.1–25.8)
Ankle	38	15.4 (10.9–20.0)	46	13.0 (9.5–16.5)	84	14.0 (11.2–16.8)
Cervical spine	21	8.5 (5.0–12.1)	32	9.0 (6.0–12.0)	53	8.8 (6.6–11.1)
1st metatarsophalangeal joint	7	2.8 (0.8–4.9)	5	1.4 (1.8–2.6)	12	2.0 (0.9–3.1)
1st carpometacarpal joint	6	2.4 (0.5–4.4)	5	1.4 (1.8–2.6)	11	1.8 (0.8–2.9)
	Women (n = 268)	Prevalence % (95% CI)	Men (n = 367)	Prevalence % (95% CI)	All (n = 635)	Overall prevalence (95% CI)
**OA (self-report physician-diagnosed)**						
Knee	31	11.6 (7.7–15.4)	54	14.7 (11.1–18.4)	85	13.4 (10.7–16.0)
Hip	21	7.8 (4.6–11.1)	45	12.3 (8.9–15.6)	66	10.4 (8.0–12.8)
Lumbar spine	20	7.5 (4.3–10.6) *	9	2.5 (0.9–4.0)	29	4.6 (2.9–6.2)
1st carpometacarpal joint	6	2.2 (0.5–4.0)	8	2.2 (0.7–3.7)	14	2.2 (1.1–3.4)
1st metatarsophalangeal joint	5	1.9 (0.2–3.5)	8	2.2 (0.7–3.7)	13	2.0 (0.9–3.2)
Cervical spine	5	1.9 (0.2–3.5)	7	1.9 (0.5–3.3)	12	1.9 (0.8–3.0)
Ankle	6	2.2 (0.5–4.0)	5	1.4 (0.2–2.6)	11	1.7 (0.7–2.8)

p = < 0.01

*Men to women

**Table 6:** Factors and prevalence of joint replacement in retired Great Britain’s Olympians

		Prevalence (%)	Univariable	Adjusted for age	Adjusted for age + sex	Adjusted for age + sex + BMI	Adjusted for age + sex + BMI + injury	Adjusted for age + sex + BMI + injury + career duration
**Knee replacement:**								
Age (years, SD)		**60.29 (15.23)**	1.06 (1.03, 1.09)***	–	1.06 (1.03, 1.09)***	1.06 (1.03, 1.10)***	1.07 (1.04, 1.11)***	1.07 (1.04, 1.10)***
Sex								
	Male	24/364 (6.59)	1	1	1	1	1	1
	Female	10/264 (3.79)	0.56 (0.26, 1.19)	0.97 (0.44, 2.17)	–	0.98 (0.43, 2.22)	1.04 (0.45, 2.39)	0.94 (0.40, 2.25)
BMI (kg/m^2^)		24.77 (3.92)	1.11 (1.04, 1.19)**	1.11 (1.03, 1.19)**	1.11 (1.03, 1.19)**	–	1.11 (1.03, 1.19)**	1.11 (1.03, 1.20)**
Knee injury								
	No	21/522 (4.02)	1	1	1	1	1	1
	Yes	13/98 (13.27)	3.65 (1.76, 7.56)**	5.17 (2.37, 11.29)***	5.17 (2.37, 11.29)***	5.04 (2.29, 11.08)***	–	5.29 (2.39, 11.74)***
Career duration (years)		10.38 (9.02)	0.98 (0.94, 1.02)	0.99 (0.95, 1.04)	0.99 (0.95, 1.04)	0.99 (0.95, 1.03)	0.99 (0.94, 1.03)	–
**Hip replacement:**								
Age (years, SD)		60.29 (15.23)	1.08 (1.06, 1.11)***	–	1.09 (1.06, 1.12)***	1.09 (1.06, 1.12)***	1.09 (1.06, 1.12)***	1.09 (1.06, 1.12)***
Sex								
	Male	30/364 (8.24)	1	1	1	1	1	1
	Female	14/264 (5.30)	0.62 (0.32, 1.20)	1.31 (0.64, 2.69)	–	1.33 (0.65, 2.73)	1.34 (0.65, 2.79)	1.29 (0.61, 2.72)
BMI (kg/m^2^)		24.77 (3.92)	1.01 (0.94, 1.09)	1.00 (0.93, 1.09)	1.01 (0.93, 1.09)	–	1.01 (0.93, 1.09)	1.01 (0.93, 1.10)
Hip injury								
	No	40/606 (6.60)	1	1	1	1	1	1
	Yes	4/13 (30.77)	6.29 (1.86, 21.32)**	8.74 (2.13, 35.89)**	8.76 (2.15, 35.66)**	8.72 (2.15, 35.43)**	**–**	8.71 (2.13, 35.63)**
Career duration (years)		10.38 (9.02)	0.98 (0.94, 1.02)	0.99 (0.96, 1.03)	0.99 (0.95, 1.03)	0.99 (0.95, 1.03)	0.99 (0.96, 1.03)	–

*BMI* body mass index

*p < 0.05

**p < 0.01

***p < 0.001

## Discussion

The aims of the study were to describe the lifetime prevalence of medical attention injuries that occurred during training and/or competition as part of the athlete’s Olympic career, and the point prevalence of pain and OA among retired GB Olympians. The main findings are that (1) 56.6% of retired Olympians self-reported sustaining at least one significant Olympic career injury; (2) each Olympian reported a mean of 1.11 injuries (females 1.37, males 0.92) during their Olympic career, with the majority of injuries attributed to training; (3) injuries most frequently occurred at the knee, lower back, and shoulder; (4) 19.5% self-reported they retired early from their Olympic careers because of injury; (5) current pain was most prevalent at the lumbar spine (32.8%), knee (25.3%), and hip (22.5%), and OA at the knee (13.4%), hip (10.4%), and lumbar spine (4.6%); and (6) injury was the main attributable factor associated with pain, OA, and joint replacement at the hip and knee.

There is a paucity of existing data for whole career injuries in Olympians, with only one global study available that was conducted as a follow-up to the current study. Using a similar injury definition, the Retired Olympian Musculoskeletal Health Study (ROMHS) reported Olympic career sports injuries in 3357 former Olympians and found a 63.0% injury prevalence [26], compared to 56.6% in the present study. This lower injury prevalence in retired GB Olympians may in part reflect a higher injury prevalence among Winter Olympians in the ROMHS cohort and a reduced risk of exposure to injury in GB Olympians due to fewer years of participating within Olympic sports (mean 9.8 ± 6.2 years), compared to the ROMHS (mean 10.4 ± 5.6 years). However, the number of injuries reported per Olympian was the same (1.1 injuries) after excluding non-sports injuries with a higher injury prevalence in training.

The results of this study expand previous observations of injury data in current athletes. The most prominent injury locations in retired GB Olympians were the knee, lumbar spine, and shoulder. These findings accord with data from retired athletes [26], and current athletes at the 2010 Winter Olympic Games in Vancouver [4]. In comparison, the thigh, knee, and lumbar spine were the most prominent injury locations at the Sochi 2014 Winter Olympic Games [27], compared to the knee, thigh, and ankle at the Rio de Janeiro 2016 Olympic Summer Games [9]. In our study population, injury was more prevalent at the knee and lumbar spine in both summer and winter sports, particularly among those competing in weight-bearing loading sports. Injury prevention initiatives should consider that sport injuries at the hip, knee, and lumbar spine were associated with a higher prevalence of morbidity in later life compared to those without injury.

The most frequent injury types were ligament sprain, traumatic fractures, and muscle injury with no differences between sexes in the type of injury except for fractures. Females self-reported a lower Olympic career BMI and approximately twice as many stress fractures compared to males. This may be a result of impaired physiological functioning caused by low energy availability (LEA) which underpins relative energy deficiency syndrome (RED-S). This syndrome can lead to impaired physiological functioning and impairments in bone health, immunity, menstrual function, protein synthesis, and cardiovascular health [28]. Prior studies have shown that specific athletic populations at an increased risk for lower bone mineral density include swimmers, runners, and cyclists [29–32]. Our study confirms a history of stress fractures among female swimmers (lumbar spine), runners (ankle and tibia), and extends this to rowers (ribs) and gymnasts (tibia). It should be noted that our study does not show causation with LEA and warrants further investigation to determine and to mitigate the risk of stress fractures in female athletic populations.

Our study illustrates that the prevalence of fractures, dislocations, severe head, and cervical spine injuries were greater among retired athletes from winter sports, particularly those with high-speed disciplines such as skiing, skating, and snowboarding. This trend is supported by data in the literature that indicates severe injuries are higher at the Winter Olympics. The number of head injuries (sport-related concussion) in 2018 PyeongChang (1.72 per 1000 athletes) and 2010 Vancouver (7.8 per 1000 participating athletes) were higher compared to 2008 Beijing (1.09 per 1000 registered athletes), 2012 London (0.57 per 1000 athletes), and 2016 Rio de Janeiro (1.06 per 1000 athletes) [3, 4, 8–10]. In total, we confirmed 10 neck injuries, 16 to the head/face, and 2 severe sport-related concussion injuries (3.08 per 1000 athletes).

Injury prevalence was highest in field athletics, gymnastics, track athletics, and lowest for canoeing, swimming, and sailing (sports with n ≥ 15 participants). During Rio de Janeiro 2016 Olympic Games, the percentage of injured athletes was also high in athletics and artistic gymnastics, and low in canoeing (slalom and sprint), sailing, and swimming [9]. In the 2012 London Olympic Games, injury prevalence was low for canoeing (slalom and sprint) and swimming, but high for athletics, synchronised swimming, water polo, and sailing [10]. The percentage of athletes injured was highest in skiing and snowboarding disciplines at the 2014 and 2018 Winter Olympic Games [8, 21].

The present study illustrates that approximately one in two retired GB Olympians reported current musculoskeletal pain, irrespective of the underlying structural changes associated with OA. Knee pain prevalence (23.4%) was lower compared to 27.3% in retired male international athletes [16], and 52.2% in ex-football players [15, 33]. Osteoarthritis was most prevalent at the knee joint (12.5%), the hip joint (10.2%), and the lumbar spine (4.5%). For the hip and knee, this is lower compared to 14.2% and 19.4% reported in 664 former male elite athletes [14], and 20.2% and 27.3% in 301 former athletes competing in power sports (boxers, wrestlers, weight lifters, throwers) [16]. The prevalence of OA was lower compared to those reported among former professional football players—18.8% at the lumbar spine [34], 13.2% at the hip joint [35], and 21.3% at the knee [36].

In our study population, injury was associated with early onset of OA in the lower limbs (hip, knee, and ankle) before 45 years and OA-joint replacement at the hip and knee. The risk of PTOA at the knee before 45 years was reported to be high among team sport athletes (soccer, ice hockey, and basketball players) [16], and soccer players and weight-lifters [17]. Our study extends this knowledge to the lower limb joints and retired athletes competing in weight-bearing loading sports including athletics (running, jumping, throwing events), hockey, figure skating, and skiing.

There are several potential explanations for differences in the prevalence of pain and OA in the reviewed literature. There are different definitions and different diagnostic criteria for OA, including radiography [15, 33, 37], arthroscopy [38], or a self-reported physician-diagnosis OA [20, 36]. There are different sports involved in the studies in the literature that may potentially affect the prevalence of OA. There are also variations in methodological procedures with some studies measuring the prevalence rate at each limb [36]. Whereas other studies measure the prevalence of OA according to the most severe limb [37].

This study examined the consequences of injury associated with retirement from Olympic sports. This study is not without limitations. Firstly, there is a possibility that retired GB Olympians who had previously sustained an injury would have a greater propensity to partake in this study. To mitigate the risk of recruitment bias, we invited all GB Olympians on the BOA Olympian database the opportunity to complete and return a questionnaire. We made strenuous efforts to mail the questionnaire to retired athletes living in 30 different countries. Secondly, the cross-sectional design may be limited by recall bias, particularly as the majority of participants had retired over 5 years ago. We attempted to offset this risk by including an injury definition that asked participants to recall only significant injuries they had sustained and were more likely to recall. Thirdly, the medical attention injury definition used in surveillance studies at the current Olympic Games may allow sports with a high frequency of minor injuries to present with a higher injury prevalence. Finally, the athlete numbers in several sports were too low to provide a reasonable comparison. Further investigation in these sports with athletes from other National Olympic Committees is recommended.

## Conclusion

The lifetime prevalence of Olympic career injury was 56.6%, with 19.5% of participants retiring early from Olympic sport due to injury. Sport injury was the main attributable factor associated with self-reported current pain and/or OA at the hip, knee, and lumbar spine, early PTOA in the lower limb (hip, knee, ankle), and joint replacement at the hip and knee. Prevention strategies to reduce morbidity and improve long-term health should aim to reduce injuries specifically at the hip, knee, and lumbar spine. Further study is recommended to determine if Olympic athletes are at greater risk of pain, OA, and joint replacement compared with the general population.

## Data Availability

An anonymised summary of the dataset generated and analysed during the current study may be available from the corresponding author on reasonable request.

## Abbreviations

GB: Great Britain
OA: Osteoarthritis
IOC: International Olympic Committee
BOA: British Olympic Association
OR: Odds ratio
aOR: Adjusted odds ratio
CI: Confidence interval
ROMHS: Retired Olympian Musculoskeletal Health Study
RED-S: Relative energy deficiency syndrome
LEA: Low energy availability
